# Indigenous birth support worker (IBSW) program evaluation: a qualitative analysis of program workers and clients’ perspectives

**DOI:** 10.1186/s12884-023-05695-2

**Published:** 2023-06-01

**Authors:** Mamata Pandey, Leanne Smith, Nicole MacZek, Angela Tomkins, JoLee Sasakamoose

**Affiliations:** 1 Research Department, Saskatchewan Health Authority, Regina, SK S4S 0A5 Canada; 2 Maternal and Child Incentive Care Unit, Jim Pattison Children’s Hospital, Saskatchewan Health Authority, Saskatoon, SK S7K 0M7 Canada; 3 Faculty of Education, University of Regina, 3737 Wascana Parkway 380 Education Building Regina, Saskatoon, SK S4S 0A2 Canada

**Keywords:** Maternal care, Culturally responsive care, Indigenous women, Cultural support, Indigenous birth support workers

## Abstract

**Background:**

The Indigenous Birth Support Worker (IBSW) Program provides Indigenous women with respectful, culturally safe, and trauma-informed care and supports women and families during labor and delivery. Located in the Jim Pattison Children’s Hospital (JPCH) Maternal Care Centre in Saskatoon, Saskatchewan, Canada, the program served 1023 clients between December 2019 and January 2021.

**Methods:**

The study objective was to explore the perspectives of the IBSWs and program clients one year post-implementation. The research plan was developed in collaboration with the IBSW program director and manager, IBSWs, and partners from the First Nation and Métis Health departments within the health region. A focus group with four IBSWs and individual interviews with ten clients who received services were conducted using a qualitative research design.

**Results:**

Thematic analysis revealed that clients greatly appreciated and respected the IBSWs’ cultural support and their compassionate, nonjudgmental, and safe care. IBSWs emphasized the importance of culturally safe and client-centered treatment, more effective pain management solutions, and that relationships with Elders and community healthcare personnel should be built and strengthened to improve pregnancy and postnatal care delivery. IBSWs desire to work with community healthcare providers to provide prenatal care and build relationships before delivery. IBSWs advocated for collaborative cooperation with community healthcare professionals and rural healthcare teams to enable a smooth care flow to and from communities.

**Conclusion:**

The Indigenous Birth Support Worker (IBSW) Program provides safe and client-centred care to Indigenous women during pregnancy, labour, and postpartum, consistent with the six principles proposed by BC perinatal services. IBSWs advocate for and assist Indigenous women in obtaining quality healthcare, provide traditional and cultural support, and positively affect mental health. However, the evaluation has revealed that healthcare provider insensitivity towards Indigenous clients persists. There is a need for greater role clarity and collaboration with healthcare practitioners to ensure evidence-based healthcare of the highest standard. This requires a commitment to addressing systemic issues and implementing broader calls to action and justice proposed by the Truth and Reconciliation Commission Calls to Action, the Missing and Murdered Indigenous Women and Girls Calls for Justice, and the United Nations Declaration on the Rights of Indigenous Peoples. The IBSW program offers vital support to Indigenous women during childbirth, but it must be viewed in the context of ongoing colonialism and the need for reconciliation and decolonization, requiring genuine collaboration with Indigenous peoples.

## Background

An external review of tubal ligation procedures in the Saskatoon Health Region, Saskatchewan, Canada, was undertaken following many claims from Indigenous women being pressured into the procedure [1]. The authors identified racism, a sense of exclusion from health decision-making, insufficient knowledge to make informed decisions, and coercion into tubal ligation procedures. Women expressed a sense of helplessness, invisibility, and an inability to advocate for their healthcare requirements. These negative experiences resulted in unmet needs, dissatisfaction with the care received, increased mistrust and discouraged people from accessing future healthcare, as prior research established [2, 3]. The Indigenous Birth Support Worker (IBSW) program was developed in response to this External Review and to honor the TRC Call to Action #22, which urges Indigenous patients to have access to Elders and cultural healing practices within Canadian healthcare institutions and for doctors and policymakers to recognize culture’s protective and healing significance. Based in Saskatoon’s Jim Pattison Children’s Hospital Maternal Care Centre, the IBSW Program provides Indigenous women with respectful, culturally safe, and trauma-informed care throughout labor, delivery and postpartum. The Saskatchewan Health Authority (SHA) and Gabriel Dumont Technical Institute coordinated IBSW training that included (1) Indigenous birth ceremony and cultural practices facilitated by an Indigenous midwife working at Sturgeon Lake First Nation, Saskatchewan and (2) a midwife in collaboration with an Indigenous midwife delivered the doula training taught according to DONA International’s educational standards. IBSWs received orientation from the Maternal Services unit. They were supervised by an Indigenous registered nurse during their practicum placement on the Maternal Service unit. Sessions on breastfeeding and nutrition were facilitated by western-trained facilitators.

The IBSW program is well aligned with these six fundamental principles proposed by Perinatal Services, BC: (1) cultural safety and cultural humility, ensuring patients feel supported and safe; (2) self-determination, providing patients options to make informed decisions; (3) trusting relationships; (4) respect, demonstrating an understanding of and respect for traditional practices and knowledge; (5) anti-Indigenous racism, raising awareness of overt and covert racism; (6) strengths-based and trauma-informed care, by focusing on patient strengths [4]. IBSWs’ values, beliefs, and practices are also shaped by their experiences as Indigenous women and mothers. They bridge the cultural gap by allowing families to incorporate traditional birthing protocols and practices into the birthing process [4, 5] to restore birth as ceremony.

## Methods

Between December 2019 and January 2021, postimplementation, 1023 clients accessed care. This study aimed to explore the perspectives of IBSWs and program clients one year post-implementation. The research plan was developed in collaboration with IBSW program director and manager, members of the SHA’s First Nations and Métis Health departments, Elders, IBSWs, and community and patient consultants. The study was approved by the SHA Research Ethics Board (REB 20–64). The study was conducted in two parts using a qualitative research design.

### Part 1

The study goals were communicated to the four IBSWs who provided services through this program during the study period. The four Indigenous IBSWs were recruited by the first author. They signed a consent form and participated in a focus group through Webex video conferencing in compliance with the pandemic protocols. Two 2-hour sessions were held and were audio recorded. IBSWs’ views on (1) services; (2) client needs; (3) barriers or challenges with access to care; (4) communication and relationships with other healthcare professionals; (5) lessons learned; and (6) recommendations for improvement were explored.

### Part 2

Between April-June 2021, clients who had delivered and were receiving services from the IBSW program were approached for individual interviews before discharge. IBSWs and the program manager assisted with participant recruitment. Questions were limited to three categories as we were sensitive to the clients’ physical and mental status post-delivery: (1) IBSW program services received; (2) IBSW and other healthcare staff experiences; and (3) client experiences and recommendations. Following the COVID-19 protocols and to ensure the confidentiality of clients who were in the healthcare facility during the interviews, multiple options were provided to allow interested clients to participate. A Regina-based researcher completed telephonic interviews with four clients. In-person interviews were carried out with three clients by the program manager. Three clients submitted handwritten responses without identifying information in a sealed nameless envelope which was collected by the program manager or the nurse on duty. Out of three additional patients who were interested, one could not participate due to health reasons, and the two other clients were discharged before interviews were scheduled. Therefore, a total of 10 client interviews were included in the data analysis. All interview and focus group data were transcribed verbatim.

### Analysis

**Fig. 1:**
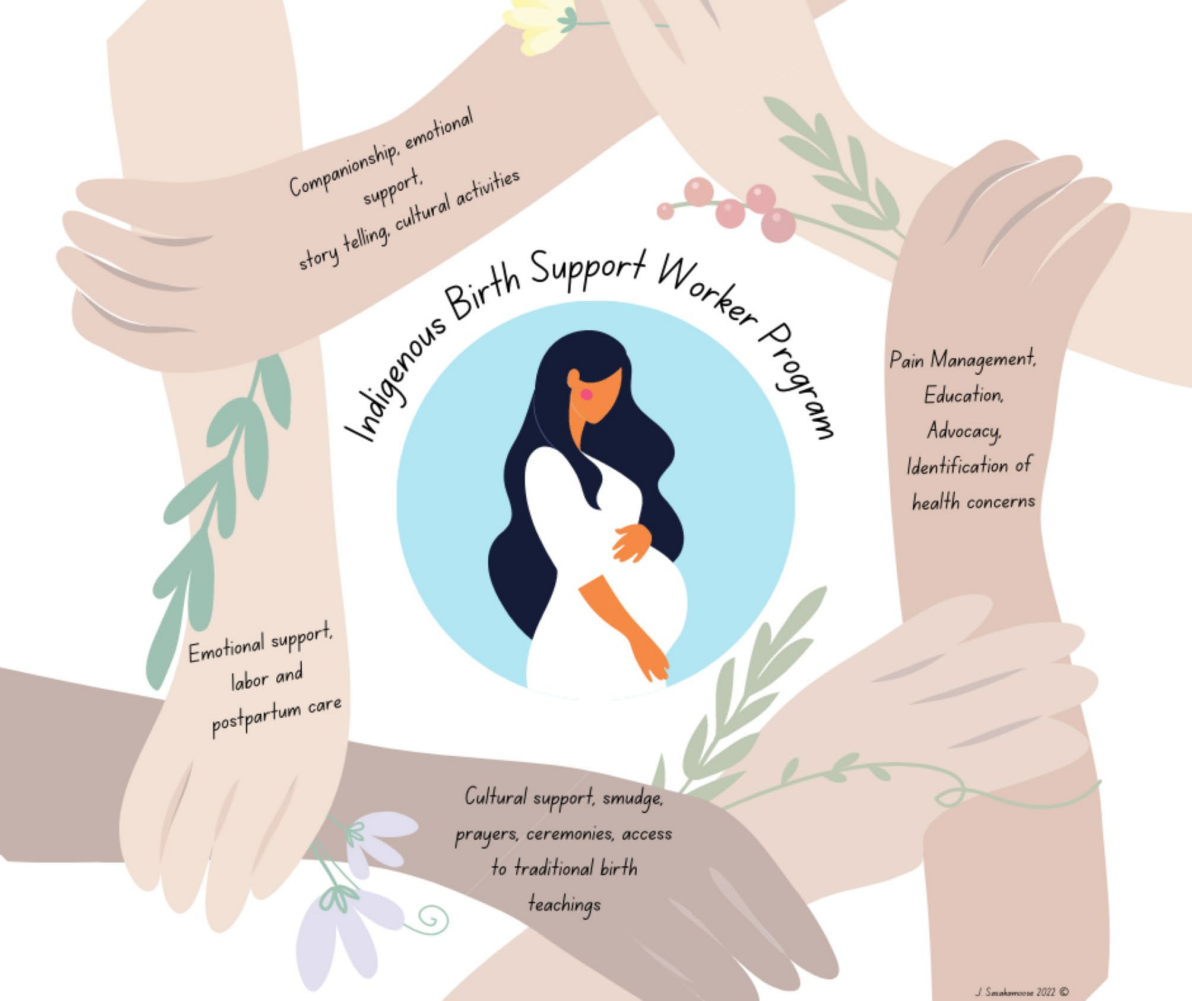
Indigenous Birth Support Worker Program

NVivo (version 9) was used to examine the data thematically. The data analysis began with two detailed transcripts, following methods described in [6]. The interview was read and segmented line by line. Each data type was assigned a title that served as the base level code, and 48 base codes were generated. This framework was used to guide the rest of the interview data analysis. Once no new topics emerged, participant recruitment was halted. Transcribed focus group material was coded into 98 categories. The 48 client interview codes and 98 focus group codes were harmonized. Sixteen intermediate themes were created from the 146 base-level codes. Each intermediate code was titled and described. The intermediate code had six themes. Diagrams and figures were employed to identify the relationships between the intermediate and primary themes. The program team reviewed the data analysis report and provided input. Figure 1 illustrates how clients and IBSWs describe the IBSW program.

## Results

### Clients’ needs

IBSWs identified their patients’ physical, emotional, cultural, and spiritual needs. Insufficient access to prenatal care in the communities and a lack of understanding of ways to navigate the urban healthcare system were also factors. An IBSW acknowledged that many of their clients are experiencing difficult circumstances and stated:

Lots of people come from very difficult situations, like domestic violence, and they’re having stressful pregnancies.

Clients and IBSWs expressed a concern that women—particularly those travelling from rural and isolated areas—may lack family support. Families frequently have work and family responsibilities and birth evacuation policies that only bring the patient, leaving them without support. Out-of-town patients may be left alone in an unfamiliar medical setting; thus, one client noted:

I have a support person here, but she’s actually back on the reserve to handle her home situation, and then she’ll be back in a couple of days.

This is also evident from another IBSW’s statement:

I also feel like the people that come from remote communities or from up north; usually get flown in for emergencies, so they’re all alone, and they stay alone. Whereas people from Saskatoon can bring support or get support to come in if needed.

As one client mentioned, health concerns, lack of family support, and unexpected surroundings can all contribute to increased stress:

It’s pretty boring in the hospital. Since I’ve been here, I lacked sleep, tossing and turning. But, I had a good sleep last night, finally.

IBSWs noted that clients’ health concerns, especially pain, may be overlooked or inadequately addressed. Both IBSWs and clients expressed concerns about ineffective pain management. An IBSW mentioned:

I think, too, that sometimes when people [patients] say something about their pain, it’s dismissed. And they’re [patient] like, “I’ll just deal with it.” So they don’t even wanna own that they are in pain… they are quiet out of fear of worse treatment.

Clients frequently required financial assistance or connections to community resources to manage additional needs such as childcare or transportation; accordingly, one client mentioned:

I need to get on the assist program or the disability program, something like that, ‘cause I don’t want to apply for the CERB anymore. So, I want to get on whatever social support system while I’m here. And, I was trying to ask people if there’s any help in the government area, see if I can get a babysitting cheque for the person babysitting [other children at home] for me.

Since clients may require assistance with childcare, housing, or other living expenses, one client described:

That was my concern, being away from home. I need some assistance to pay my rent and then help my sitter out with the groceries and stuff.

When clients are physically and mentally vulnerable without adequate support, daily living necessities can be more demanding. One of the clients indicated that cultural support would be valuable during difficult times:

I would love more information and for [ceremonies] to be happening, for that to be arranged. I am going through a lot, and having some Elder support would be very effective for helping me get through this time.

IBSWs stated that they provide various services during pregnancy, labor, and postpartum. The clients are also referred by healthcare professionals familiar with the IBSW program. Prenatal education, birth plan development, labor and delivery support, nonpharmacological pain management, postpartum education and support, breastfeeding assistance, and other services. As an IBSW commented:

It depends on when they come into the hospital. Some women come in as prenatal. Sometimes we catch ladies when they’re laboring and agree to have our support during their labor. So we’re there to help keep them calm and guide them through their labor. And even post-partum, sometimes we don’t meet the ladies until they’ve already had their babies. We’re there to help them with breastfeeding.

IBSWs provide companionship and emotional support by creating a safe space and practicing empathetic listening. As one IBSW noticed, many clients may have experienced challenging life situations and require additional support, as reflected by this comment:

When we work with prenatal patients, one of the biggest things we do is offer emotional support. People often come in from very difficult situations requiring someone to listen. And I feel like, with us, they’re more open. We just tell them, “I’m not going to judge you. You can tell me whatever you need to. I’ll help, however, I can.” They confide in us so much. So, lots of emotional support, lots of smudging.

The clients expressed gratitude for the emotional assistance received during stressful times, particularly during the COVID-19 pandemic, when family visits were limited and family support, which is generally offered to most clients, was absent. A client mentioned:

It’s just hard. But the support worker last night, she stayed with me late, keeping me company. We talked, and she was very helpful.

IBSWs visited patients frequently throughout their hospital stay, educating them and entertaining them. This was helpful for out-of-town patients without family support. According to an IBSW:

We all try to pop in every day, on a shift, to go see them, say hello. Introduce ourselves; We see if they need anything, like snacks or drinks. Ask if they want to try any of our activity stuff we have ‘cause we have adult calming coloring books, word search, and a bunch of things they could do. A lot of our kits are geared towards the prenatal to prepare for their babies.

The patients also valued the one-on-one attention and assistance they received from IBSWs, particularly during the COVID-19 pandemic, as evidenced by a client’s comment:

*The girls [IBSW] are super friendly and easy to get along with, so that was nice. My husband wasn’t able to be in the hospital much. So, they came in, so I wasn’t alone as much. And they had taught me how to bead, so that was nice to have something to do while I was just sittin’ around. Then they would run and grab me snacks and stuff if I needed it*.

As demonstrated by this comment made by an IBSW, respectful, culturally safe, and trauma-informed care improved the quality of the patient experience:

*“I found them supportive, received a moss bag and beaded earrings. I came to the hospital in shorts and didn’t have anything. They brought me shoes and shampoo. I came to the hospital alone, so it was nice to have them. They were supportive, friendly and made me feel comfortable. It was great that IBSW took me upstairs (NICU) to see my baby right after delivery. She didn’t have to, but she did. I enjoyed my time with them.”*

As evidenced by the IBSW’s response, they prioritized the client’s physical and mental health and respected their decision to seek services.

I think it depends on their personal personality. Some people want to keep their birth process private, with just them and their spouse. So we’re fine with that.

IBSWs also knew that many clients had previous negative experiences with the healthcare system and felt vulnerable. IBSWs asked permission before providing services, respecting clients’ rights to choose the services they wanted. According to an IBSW, services were tailored to the clients’ needs while giving trauma-informed care:

We introduce ourselves. Tell them we’re on for our shift, and we’ll pop in now and then if they allow us to. Again, we always ask for permission. I think that’s really important.

IBSW observed that many clients were unaware of their rights or their eligible services. IBSWs frequently assisted clients in obtaining additional services they required, as demonstrated by the following client comment:

Also, they put me in touch with a couple of people able to come and advocate for me, on my behalf, with the CFS, to help me get my daughter back into my care. So that was really good. They’ve been helpful for everything.

IBSWs are familiar with the negative encounters, racism, and substandard care encountered by many Indigenous women in healthcare settings and recognize that clients may be reluctant to express their needs. IBSWs ensured that clients felt safe and received the required care during hospitalization. An IBSW provided the following account:

*A lot of Indigenous women are just happy there’s added support for them. Because many Indigenous women, from anywhere, have often felt displaced in the medical and hospital settings. Many have experienced prejudices towards them, mistreatment, or just not feeling safe within the healthcare system.*

As attested by the following comment, clients expressed relief at the prospect of receiving assistance from IBSWs if their family members could not be present during delivery:

*It was nice to know that if my husband couldn’t come in, to make it had I gone into labor. If he couldn’t make it in time, there would be someone I already had a relationship with who could come in and be a support if he couldn’t be there. That helped my mental health, for sure.*

IBSWs understood the importance of establishing trustworthy relationships with clients to comprehend their needs and provide necessary services. IBSWs seek to provide a welcoming and nonjudgmental environment for clients and their families. As Indigenous people, IBSWs are familiar with cultural protocols and the importance of establishing relationships to foster trust with clients and their families, as demonstrated by this comment:

*“When I go into a room and introduce myself, I often try to be a part of their family. I just say, think of me as your sister, your aunty, your Kookum, your friend.” And I do whatever I can to make their stay comfortable and safe.”*

IBSWs ensured that clients’ healthcare concerns were adequately addressed. IBSW staff educated patients on how care is coordinated and connected them to appropriate hospital and community services. They advocated for their rights and ensured that mistreatment or substandard care incidents were addressed. This is clear from an IBSW’s statements:

If they felt like they were mistreated during their experience in the hospital, we let the managers know so they could further investigate and talk to those patients about why things were done the way they were done. So, they’re not being sent out traumatized from their experience in the hospital.

IBSWs informed clients about postpartum health warning signs and urged them to notify healthcare providers if their health concerns worsened. IBSWs aided clients in managing their pain and advocated for their healthcare needs, ensuring they were met promptly. This is demonstrated by the following comment made by an IBSW:

I have gone to a nurse, saying, “This patient is in pain, but she’s scared to ask for any medication because she feels like there are prejudices against her.” And the nurse is fine with checking her over and getting her some pain medications she needed. But some women are too timid to even ask because many women are labelled [as drug seeking].

When clients collaborated with IBSWs, they felt a sense of security. IBSWs assisted clients in resolving their concerns when they expressed dissatisfaction with their treatment or believed they were being mistreated, as evidenced by the following client comment:

I have had been experiencing some stigma, specifically last night, and I made a complaint, and a different doctor was put to take care of me instead of the other one. But the support worker helped advocate that for me and helped me make that complaint. And I don’t think I would’ve if she wasn’t there.

IBSWs assisted clients who may require additional assistance in monitoring their health status, advocating for themselves in the healthcare system, and receiving timely care.

### Cultural support

IBSW program components include culturally responsive treatment, access to Indigenous health, traditional birthing practices, and education. IBSWs provided Elder connections and cultural access, such as smudging. IBSWs arranged Elders, prayers, and ceremonies as requested by clients. They use storytelling to connect clients to traditional preconception, pregnancy, labor, and postpartum teachings. Patients could participate in beading, artwork, and traditional crafts. Traditional arts were introduced to help patients avoid undue stress during hospitalization. The artwork is a memento the mother can take home after childbirth. IBSWs connected clients to their cultural background through activities, reintroducing birthing as a ceremony. An IBSW illuminated:

We try our best to gift belly button pouches; any Indigenous person with a baby is gifted a pouch to keep the umbilical cord. It’s a small thing that might seem like- it doesn’t mean much to anybody else, but it’s part of you and your baby. It’s a sacred item to look after. We’re trying to make that more mainstream here. We try our best to touch base and give them those pouches.

These activities were well received by clients, who valued the cultural education and personalized gifts made for them and their infants, as evidenced by the following client comment:

I received a moss bag and beaded earrings. I really like the Indigenous support. Mom is Cree, and Dad is Dene. I really liked the moss bag. I liked that it was made for me, and they helped me understand its meaning as I didn’t know what it meant, but I knew I would want one for my baby someday.

IBSWs recognize the rich and diverse cultural heritage of Indigenous peoples. They provided cultural support and services but acknowledged that they lacked knowledge and understanding of the various Indigenous cultures and languages. While they could offer prayers and smudging, they respectfully informed clients they lacked the same knowledge and wisdom as community Elders and Knowledge Keepers. The IBSWs’ perspectives are reflected in the following comments:

There are so many tribes. There’s Cree, Dene, Saulteaux, and Dakota. We don’t know everybody’s specifics except for our own. We’re not all fluent in our languages; we’re all learning. And I can’t do a full-on prayer [in the language]. I can’t provide them with spiritual comfort like that. Yes, I can bring in smudge; I can sit there and smudge with them, pray in my mind. But I can’t do all the things an Elder can do.

As an IBSW remarked, cultural services are frequently sought by clients. “I have gone into rooms, brought moms to smudge occasionally, and they ask, “*Can you say a prayer for me?“* Clients who received such assistance expressed gratitude acknowledging that it made them feel better; as one client stated, *“I smudged today, and it made me feel better.“* Clients and families felt that their culture and traditions were recognized and respected, as evidenced by a client:

I like she’s thorough. She makes sure that we have the information we need. I like that she gave us pamphlets on the different traditional teachings and the little belly button pouch. And it was comforting to know those were offered services here at the hospital, ‘cause I felt like it represented us as a family and what we would want in our baby’s care.

While promoting the culture, IBSWs had to navigate the tensions between evidence-based Western practices and Indigenous ways of knowing, as an IBSW revealed.

*“Fathers have asked me, ‘Can you teach me how to make a moss bag?‘ I’ve also instructed the fathers on how to construct an Indian swing, as this is another of our teachings. However, we should not promote it because it can result in liabilities within Western care models. However, we have followed these practices for hundreds of thousands of years. Therefore, it’s not that bad. I always inquire; I always attempt to include men. And if a sister or Kookum is involved, I incorporate their ideas. Typically, they already know, so they are already performing the tasks we assist with. It’s wonderful to observe that.”*

IBSWs and clients reported that family participation was reduced due to COVID-19 safety protocols, which had a detrimental influence on the client’s well-being because assistance from family members was unavailable. Lack of support can be especially traumatic with prenatal bereavement, as evidenced by a comment made by an IBSW:

COVID changed a lot of things for how our people like to celebrate and how we also mourn when there’s a loss. ‘Cause it’s big to have your support there. Even with a delivery. Even with a loss. It balances itself all out with all that stuff when you have your family and friends there to support you.

### Experience with healthcare

IBSWs found that clients frequently struggle to communicate their needs to healthcare practitioners and that their requests are commonly ignored or unmet. As an IBSW reflects:

There have been prenatal patients who come in with a lot of pain, and they feel like they don’t want to ask the nurses for pain medication ‘cause they feel like they’re being labelled as drug seekers, but they don’t know the cause of their pain until it’s diagnosed. So, a lot of times, some women didn’t feel… safe or heard in telling their nurses.

IBSWs observed poor pain management or delays in treating health conditions, which can result in more intensive procedures later or become potentially life-threatening, ending in suboptimal care for the patient, as illustrated by an IBSW:

*She [patient] was suffering from a hematoma. So, it’s like an internal hemorrhage that could’ve been caught sooner. However, luckily, they controlled it in the OR. So, her and her partner were complaining about that ‘cause they didn’t feel they were treated fairly. They felt like the nurse was rude to them.*

Clients also mention instances when their pain was not addressed adequately, increasing their discomfort, making them feel helpless, and minimizing their concerns leading to suboptimal care and dissatisfaction. This is evident from a client’s comment:

She [doctor] wasn’t taking me seriously when the doctor came back in; she was being very condescending about my pain. And I, again, would try to make eye contact with her, try to explain, “Yes, I’m still in pain. I’ve told you this often now.” And she wasn’t giving me a good standard of care. But I was getting very hurt because it felt like she was intentionally not taking what I was going through seriously.

Other clients were reportedly satisfied with the care received, as evidenced by a client’s comment: *“Everybody has been very kind and let me know, communicating properly and everything. It’s been good so far.”* Another client mentioned, *“Some of the nurses I have are good, and they check in frequently.”*

IBSWs mentioned that sometimes the client’s involvement was minimized during labour and delivery. Clients often felt ignored and excluded, leading to dissatisfaction with the care received. This was evident from the IBSW comment:

*Just making them feel important. ‘Cause sometimes it’s just like, Hi, I’m blah, blah,” and then deliver the baby and they’re [healthcare provider] gone. “Okay, I’m onto the next delivery! So that connection with them is important. It’s just validating that woman’s presence. She’s there to have the baby, but she’s also there- she’s gonna remember everything here. They [patients] feel like they blend in with everything else like they’re not important enough.*

All clients were very satisfied with the care, support, and services received from the IBSWs. One client mentioned, *“I enjoyed my time with them; it’s good; they were the most help out of everyone.”* Other clients commented that the IBSWs were approachable and compassionate, as is evident from this client’s comment: *“They’re really helpful, and they’re supportive. They check on me daily and make sure that I’m doing okay, they’ve been very helpful during my stay here.”*

### Communication

IBSWs stated that many healthcare providers were uninformed of the IBSW practice. Occasionally, IBSWs were mistaken for nursing personnel or social workers, causing confusion, as evidenced by an IBSW comment:

I’ve been confused with- I’d be in with a client, in the room, and a doctor would walk in and ask the questions she needed to, and then looked at me and said, “Did I do everything? Is there anything else?” I always have to say, “No, I’m just the IBSW.” And then she’s like, “Okay,” and then leave the room. So not everybody knows who we are.

According to IBSWs, sharing information on Indigenous birth practices and the procedures they follow will increase the understanding of Indigenous birth traditions among healthcare teams. IBSWs assert that this will ensure the reliability of those practices and teachings. As evidenced by this IBSW’s remark, improved communication between healthcare teams and IBSWs will increase referrals, uptake, and the quality of care provided:

We do have a strict way of doing things. .. the teachings of our knowledge. I think that’s where the knowledge is lacking. If the staff have questions or if they don’t know what we’re doing is right, they can ask us or our knowledge keepers, who have trained us, for more information. Just so we can get that ground-level understanding and not second-guessing one another, we can work as a team to deliver that care.

IBSWs successfully created a trusting relationship with their clients, especially while providing prenatal support, which is beneficial when clients return for delivery. The following comment from an IBSW demonstrates this:

We have seen a few where they’ve been prenatal for a while; we’ve all gotten into contact, all got to visit with this certain individual. They had their baby post-partum care. They came back again because of issues with the surgery they had. They look forward to seeing us. And I look forward to seeing them, even though the circumstances aren’t great for her to be back here. She knows we’re there. I think they appreciate that too.

Clients expressed satisfaction with their interactions with the IBSW, who was approachable, empathetic, and assured that the clients’ needs were addressed, as indicated by these client’s comments:

*“They come and talk if I want to get something off my chest; they want to talk. They let me know they’re there whenever I need them. I feel like it’s just warm welcoming with them. All of them came and introduced themselves.*

### Recommendations suggested by IBSWs and clients

IBSWs proposed notifying clients about the program and establishing a link before arriving at the hospital. This will particularly benefit women travelling from remote areas. A client reflected:

*I was actually surprised there was a program happening here. And then I thought it was a great idea to have that. I asked if there were any more programs, maybe in the north or elsewhere. And it’d be cool if there was. I think its really good, you guys are* doing that.

IBSWs say many Indigenous communities have limited prenatal and other healthcare services. Due to lack of transportation, childcare, and racism, some urban residents cannot attend prenatal classes, which can prepare them for delivery and parenthood. IBSWs acknowledged the importance of prenatal education for women in isolated and rural regions. They proposed that engaging with women earlier in pregnancy could provide opportunities to discuss previous trauma and healthcare concerns and build rapport. This will guarantee that women are supported and feel more comfortable in hospital settings. An IBSW noted:

It would be nice to get to know them during prenatal. So, if they have past traumas, to help them heal from so their birth experience can become pleasant, instead of always going into it scared and traumatized.

Indigenous peoples are a diverse group with distinct languages, traditions, and cultural practices. IBSWs who know their own culture may lack the information to provide specific spiritual support to women from other backgrounds. Elders from their respective communities are needed to conduct ceremonies and prayers and spiritually guide women. IBSWs recognized that through mentorship from Elders, they could adequately support the clients. A female Elder was needed to assist pregnant and postpartum women. An IBSW expressed:

To have an Elder come in, as we’re not all fluent in our languages; we’re all learning. And I can’t do a full-on prayer [in the language]. And I can’t provide them with that spiritual comfort like that. But I can’t do all the stuff an Elder can do. We’re not qualified [Elder] for that. And a lot of times, women need that. Our women need that here. It doesn’t matter if they’re Dene. It doesn’t matter if they’re Saulteaux. Cree. Sometimes just having that person [Elder] there to do that as a spiritual advisor is super important for us.

According to IBSWs, clients often seek help from Elders or spiritual advisors during prenatal loss. Elders’ support is critical in providing spiritual direction, consolation, and healing and was not available in the IBSW program: *“during losses, some people want a priest, or they want an Elder, and we don’t have an Elder to provide women when going through miscarriages or anything.*”

IBSW underlined the need to build relationships with communities and community birthing knowledge keepers so that women, particularly those from isolated and rural communities, feel comfortable and protected while in the hospital. Creating relationships can help clients adjust to their new surroundings. An IBSW illustrated:

If we integrate with the community, their Elders, and their ways of knowing...Communicating with traditional midwives in communities or birth doulas and allowing the space to connect and work with them and build that relationship so we can call upon them. When a community member comes into our space, we can have that communication and that relationship with the community; I feel like it would benefit the women. So they don’t feel like they’re in a foreign place, away from their people. And they’re seeing new faces; they don’t know if they can trust or open up to seek services. Because a lot of them are closed off in that way.

IBSWs indicated a need for improved communication and training of health care providers and client education about pain management. IBSWs confirmed that clients encountered discrimination, substandard care, and dissatisfaction with pain management. As is evident from this comment:

They just felt like they were being labelled when something was going on, and they were in pain... they felt that the nurses treated them wrongly or like their pain wasn’t valid. So those have had to be dealt with.

IBSWs suggested increasing healthcare professionals’ and clients’ awareness of the program’s scope and services and improving system integration.

## Discussion

The study results indicate that the client-centered, culturally responsive, trauma-informed care provided by the IBSW program was well received by Indigenous women receiving maternal care in the hospital setting. Clients and their families appreciated the cultural support and traditional guidance provided through the programs. Clients requested Elder services, spiritual care, and access to culture throughout pregnancy, childbirth, parenthood, and perinatal loss. Indigenous women need practical pain management strategies during labor and delivery. A strong partnership between IBSWs and mainstream healthcare providers can further enhance maternal care delivery for Indigenous women.

### Context, challenges, racism, and barriers

Recognizing and valuing pregnancy and birth as sacred and natural rites of passage for all families will benefit Indigenous peoples and healthcare providers [7]. Indigenous peoples thrived before colonization because their cultural norms, beliefs, and practices improved their well-being [4, 8]. Many people still follow their family and community traditions. In Indigenous cultures, the child is the center of the family and community, and traditional practices were built around this core belief to ensure the child’s health [9]. Every family or community member played a role in daily life, whether gathering food, collecting wood for the fire, facilitating ceremonies, or imparting teachings. Ceremonies kept people mentally, physically, and spiritually strong. People felt a sense of belonging and purpose because ceremonies and *muskiki* (“medicine,” Cree) or protective factors are part of the culture. A Cree baby swing, for example, was known for balancing the baby’s inner ear fluid, preventing ear infections. Indigenous cultural aspects should be recognized and incorporated into modern medical procedures.

Indigenous cultural practices and traditions that restore birth as a ceremony are vital to the future well-being of Indigenous peoples [5, 10]. Traditional activities such as storytelling can pass on health and healing information, prepare new parents for childcare, and preserve good health [4, 11–14]. Fathers and other family members can make clothing, baby swings, and other cultural items. Fathers in the IBSW program beaded and made a moss bag to care for and support the mother and the infant. A father’s engagement during pregnancy and childbirth improves newborn feeding and boosts his satisfaction [15–17]. These activities help the mother and her newborn infant create a caring attachment, enhance family relationships, and establish a supportive family and community network. Coping with perinatal loss and grief and celebrating the new baby’s arrival in the community requires community support and ceremonies. Because ceremonies are sacred and provide individuals with a sense of connection and purpose, Indigenous health practices and cultural teachings can offer better understanding and alternative solutions for women and families facing challenging situations [11]. Through the IBSW program, women who were disconnected from their cultural traditions because of the Canadian government’s assimilation policies had access to traditional teachings that restore birth as ceremony. Mothers who required support to honor the sacred aspect of birth as a ceremony relied on the IBSWs to advocate to assure access to traditional practices and protocols.

Although Western medicine has reduced maternal and infant mortality rates in developing countries, some researchers are concerned that childbirth has become overly medicalized [18], and indigenous scholars are advocating for reproductive justice [19]. Regardless of Western interventions, poor birth outcomes among the First Nations, Métis, and Inuit people have persisted [4, 17, 20, 21]. Indigenous populations have the highest rates of larger for gestation age, and sudden infant death syndrome (SID) is seven times higher [17]. SID accounts for 24% of First Nation and 21% of Inuit infant deaths, compared to 7% for non-Indigenous individuals [21]. Colonization, residential schools, racism, systemic mistreatment of Indigenous people in healthcare settings, and racist policies are frequently cited as reasons for poor health outcomes in Saskatchewan and across Canada [3, 22].

Biaggi et al. [23] observed lack of partner or social support, history of abuse or domestic violence, personal history of mental illness, unplanned, unwanted pregnancy, adverse life events, high perceived stress, present and past pregnancy complications, and pregnancy loss as risk factors for women’s antenatal anxiety and depression. In 1892, the Canadian government implemented the Birth Evacuation Policy, which forced pregnant Indigenous women to give birth in urban hospitals. This strategy has failed to provide high-quality care. Birth evacuation affects fetal neurodevelopment, breastfeeding, and family relationships [19]. According to studies, IBSW patients in rural or remote First Nation communities receive insufficient prenatal care [3]. Many experience health issues during pregnancy or childbirth, requiring them to be transported to urban hospitals. When women give birth in unfamiliar circumstances, they are more likely to have premature births, neonatal difficulties, postpartum depression, difficulty breastfeeding, family strain, attachment disorders, and an inability to appreciate a new birth [2, 3, 24]. COVID’s visitation restrictions increased the mental health stress of the mothers, and IBSWs observed that clients’ home lives commonly harmed their mental health and lacked healthcare advocates. Indigenous women are more prone to face perinatal mental health difficulties such as depression, anxiety [25], and drug misuse; hence, the IBSW program assists with pregnancy, birth, postpartum, miscarriage, loss, abortion, and parenting [26, 27].

IBSWs reported that clients were hesitant to approach healthcare providers with health concerns, particularly about pain, fearing being viewed as “drug seeking.” Indigenous people lack access to adequate pain medications because physicians are hesitant to administer pain drugs to patients due to common misconceptions about addiction [28]. According to the literature, negative healthcare experiences discourage people from reporting problems [29, 30]. Studies suggest that pain management is insufficient for oppressed populations such as Indigenous people, refugees, and the LGBTQ community [30, 31]. As observed in another study, clients and IBSWs mentioned that pain management was inadequate or unsatisfactory [30]. Additional research suggests that skepticism may cause Indigenous people to delay seeking healthcare, leading to deteriorating health and invasive procedures [32, 33]. Fenwick [32] advocated using culturally sensitive techniques to assess Indigenous pain, arguing that failing to understand the social-cultural factors that influence pain experience and description should not be excused.

Consistent with Boyer and Bartlett’s External Review [1], clients in this evaluation felt unheard and disregarded, leading to dissatisfaction with care and deepening the distrust of the Western healthcare system. The deterioration of health conditions in ERs and acute care hospitals caused by racism and substandard care has been documented in numerous investigations across Canada [30, 33–35]. According to CBC Manitoba, Brian Sinclair died of racism after being left in the ER for 34 h. National headlines such as these reinforce that the healthcare system is unsafe for Indigenous women and the birth of their children. Systemic discrimination exists in the health care system, and health providers need trauma-informed training to increase awareness of access barriers and challenges [3, 28, 36–38]. Varcoe et al. [39] attributed the decline in Indigenous women’s and pregnant people’s perinatal health in Canada to dehumanizing interactions with healthcare providers, loss of autonomy, and discrimination and racism. Vedam et al. [40] identified abuse, such as being shouted at or scolded by healthcare providers, privacy violations, and threats to withholding treatment or coercing a patient to accept it. Indigenous women, women of color, and women with social, economic, or health issues were more likely to be abused.

The IBSW program addressed the first principle suggested by the BC perinatal services by improving access to cultural care for clients and enhancing cultural safety and humility. Furthermore, by advocating for patients and their healthcare needs, IBSWs ensured that patients at the hospital felt supported and safe. The second premise was met by IBSWs by providing service options available through the IBSW program and by following patients’ and families’ lead in selecting the appropriate cultural services. However, it is unclear whether the IBSW program itself improved patients’ and families’ ability to choose and or determine western care that was available to them. IBSWs addressed the third principle by engaging empathetic listening, and collaborating with patients to build trust. The fourth BC principle was addressed by recognizing the rich and diverse cultural backgrounds of many First Nations. IBSWs attempted to provide cultural care within their area of competence and connected with Elders, and Knowledge Keepers as needed and when available. The fifth principle was addressed by IBSWs by advocating for patients and families, raising awareness when needs were not being met, and reporting instances of inadequate, unsafe treatment. Finally, the IBSW program improved strength-based and trauma informed treatment by meeting patients where they are and offering assistance as needed to improve the quality of care provided.

According to the study findings, the IBSW program is an essential step towards addressing healthcare delivery gaps and promoting patient-centred and culturallysafe care in hospital settings. The study emphasises the importance of focusing on ongoing racism, and discrimination in healthcare settings, which impedes Indigenous patients’ self-determination, right to make informed health decisions and timely healthcare access. Despite the IBSWs vigorous campaign for their patients, initiating attitude changes among healthcare personnel and improving their cultural competency, as well as addressing systematic discrimination was out of the scope of this program.

While this evaluation demonstrates that the IBSW program achieved its objective of providing culturally responsive services to patients and families, many experiences expressed by clients reveal significant insensitivity to clients’ concerns and challenges by the existing healthcare system and care providers as documented in the literature [1, 30, 33–35]. The underlying difficulties encountered by many mothers in response to their healthcare professionals are unaffected by the number of caring, supportive people provided to clients. These challenges are expressed in comments such as the client’s needs were ‘ignored or unmet,‘ they were unheard or disregarded. Mothers found it challenging to communicate with their care provider because the provider was rude (resulting in complications during birth that were not reported earlier in labour), their needs for pain medication were not taken seriously, they felt ‘not important enough,‘ and providers appeared unaware of the IBSW’s role. However, the evaluation has also revealed that more needs to be done to address the underlying issue of healthcare provider insensitivity towards Indigenous clients. To ensure that the care provided by IBSWs is evidence-based, of the highest possible standard, safe, and rapidly attends to the needs of Indigenous women, there is a greater need for role clarity and collaboration with healthcare practitioners. This will require a commitment to addressing the systemic issues of healthcare provider insensitivity towards Indigenous clients and to implementing the broader calls to action and justice proposed by the Truth and Reconciliation Commission Calls to Action, the Missing and Murdered Indigenous Women and Girls Calls for Justice, and the United Nations Declaration on the Rights of Indigenous Peoples.

The IBSW program offers vital support to Indigenous women during childbirth, but it must be viewed in the context of colonialism’s ongoing legacy and the need for reconciliation and decolonization. Working collaboratively to address systemic concerns and implement broader calls to action and justice is critical to achieving evidence-based healthcare of the highest possible standard, while also being safe and responsive to the needs of Indigenous women. This necessitates a commitment to reconciliation, decolonization, and genuine collaboration with Indigenous peoples.

### Recommendations

Integration of the IBSW program within the healthcare system and information sessions about the IBSW program among healthcare providers can enhance program referrals and uptake. Prenatal programming delivered in collaboration with the home community can strengthen trusting and supporting relationships between First Nation communities and healthcare practitioners, facilitating care coordination between the community/rural setting and the urban hospital environment. Training for IBSWs is needed in historical trauma, trauma-informed care and practices, adverse childhood experiences (ACE), health, safety, and cultural knowledge, ensuring care delivered is high quality. Trauma-informed strength-based training for mainstream healthcare providers can help them understand their role in Indigenous well-being and promote the delivery of safe care.

The Indigenous Birth Support Worker (IBSW) Program provides valuable support to Indigenous women during childbirth by offering respectful, culturally safe, and trauma-informed care. However, as we have learned from the feedback, the program may not be enough to address the systemic issues of healthcare provider insensitivity towards Indigenous clients. Therefore, it is essential to explore additional interventions that could be implemented alongside the IBSW program to address the root causes of this problem.

One possible intervention is to provide cultural safety training to healthcare providers. This training can help providers understand the historical trauma and ongoing impacts of colonization on Indigenous peoples, which can help them provide more respectful and culturally safe care to Indigenous clients. Cultural safety training can also help providers recognize and address their biases and assumptions, which can contribute to the insensitivity experienced by Indigenous clients. Another possible intervention is to increase the number of Indigenous healthcare providers. Indigenous healthcare providers can provide more culturally safe care and be responsive to the needs of Indigenous clients. Increasing the number of Indigenous healthcare providers can also help address the systemic underrepresentation of Indigenous peoples in the healthcare system. In addition, it is essential to address the structural barriers that prevent Indigenous people from accessing culturally-responsive healthcare services. This includes attending to issues such as transportation, cost, and availability of services in remote and rural communities. Addressing these barriers can help ensure that Indigenous clients have access to the care they need and deserve.

While the IBSW program provides valuable support to Indigenous women during childbirth, more must be done to address the systemic issues of healthcare provider insensitivity towards Indigenous clients. By exploring additional interventions such as cultural safety training, increasing the number of Indigenous healthcare providers, and addressing structural barriers to healthcare access, we can work towards a healthcare system that is more respectful, responsive, and equitable for Indigenous peoples.

### Limitations

Face-to-face interviews with patients were not permitted due to COVID restrictions. Several interested participants could not be reached after discharge. Only clients in the hospital during data collection were included, resulting in selection bias. As clients were interviewed while they were still receiving care in the hospital it is possible that they might not have been able to express their view adequately. Additionally, recognizing the fragile mental and physical state of the clients the interview questions were focused on their experience with the IBSW program and no details about their delivery or demographic information were requested.

## Conclusion

The Indigenous Birth Support Worker (IBSW) Program provides safe and client-centred care to Indigenous women during pregnancy, labour, and postpartum, consistent with the six principles proposed by BC perinatal services. IBSWs advocate for and assist Indigenous women in obtaining quality healthcare, provide traditional and cultural support, and positively affect mental health. However, the evaluation has revealed that healthcare provider insensitivity towards Indigenous clients persists. There is a need for greater role clarity and collaboration with healthcare practitioners to ensure evidence-based healthcare of the highest standard. This requires a commitment to addressing systemic issues and implementing broader calls to action and justice proposed by the Truth and Reconciliation Commission Calls to Action, the Missing and Murdered Indigenous Women and Girls Calls for Justice, and the United Nations Declaration on the Rights of Indigenous Peoples. The IBSW program offers vital support to Indigenous women during childbirth, but it must be viewed in the context of ongoing colonialism and the need for reconciliation and decolonization, requiring genuine collaboration with Indigenous peoples.

## Data Availability

The datasets generated and/or analyzed during the current study are not publicly available due to the participants’ confidentiality, but are available from the corresponding author on reasonable request.

## Declarations

### Declaration of Helsinki

All methods were performed in accordance with the relevant guidelines and regulations with the Declaration of Helsinki.
